# A case report of endovascular treatment with blunt cerebrovascular injury

**DOI:** 10.1016/j.ijscr.2024.110488

**Published:** 2024-10-22

**Authors:** Xizhu Huang, Xianglei Kong, Yongxia Cai, Wenlong Fan

**Affiliations:** a Department of Emergency, Sir Run Run Shaw Hospital, Medical School of Zhejiang University, Hangzhou 310000, China; b Department of Radiology, Sir Run Run Shaw Hospital, Medical School of Zhejiang University, Hangzhou 310000, China

**Keywords:** Case, Blunt cerebrovascular injury, Severe head trauma, Hemorrhagic shock, Digital subtraction arteriography (DSA), Endovascular treatment

## Abstract

**Introduction and significance:**

Blunt cerebrovascular injury (BCVI) is a rare but potentially destructive complication following trauma.

**Case presentation:**

A patient who suffered blunt cerebrovascular injury due to trauma, leading to shock, cardiac arrest, and respiratory failure. Successful cardiopulmonary resuscitation was performed, and digital subtraction angiography (DSA) revealed active bleeding. Interventional embolization was employed to control bleeding, but the outcome was unfavorable due to ischemic-hypoxic brain injury.

**Clinical discussion:**

Careful and meticulous evaluation of the patient's condition is necessary to avoid misdiagnosis and ensure timely intervention. Delay in diagnosis results in higher mortality rates and more severe ischemia and hypoxia of brain cells, leading to worse prognoses.

**Conclusion:**

In emergency, digital subtraction angiography (DSA) combined with endovascular treatment contributes to reducing the mortality associated with this type of injury.

## Highlights

• Blunt cerebrovascular injury (BCVI) is a lethal event.
• BCVI are extremely serious, with low survival rates and a propensity for diagnostic oversight, leading to treatment delays.
• For BCVI, the urgent use of digital subtraction angiography (DSA) combined with endovascular therapy is highly necessary and effective.
• The outcome of this case was unfavorable, and the lessons learned from the failure are worth contemplating.

## 1 Introduction

Blunt cerebrovascular injury (BCVI) is a rare yet potentially devastating complication following trauma, accounting for approximately 1.5% to 3.5% of blunt trauma patients admitted to trauma centers [[2], [3], [4]]. The incidence of BCVI in patients suffering from blunt trauma is low, and the clinical presentation of BCVI varies widely depending on the affected vessels, the site of injury, the level of injury, and any pre-existing cerebrovascular disease. This frequently leads to underdiagnosis, and failure to timely identify and treat BCVI may result in serious complications and deaths.

## 2 Case presentation

**Fig. 1:**
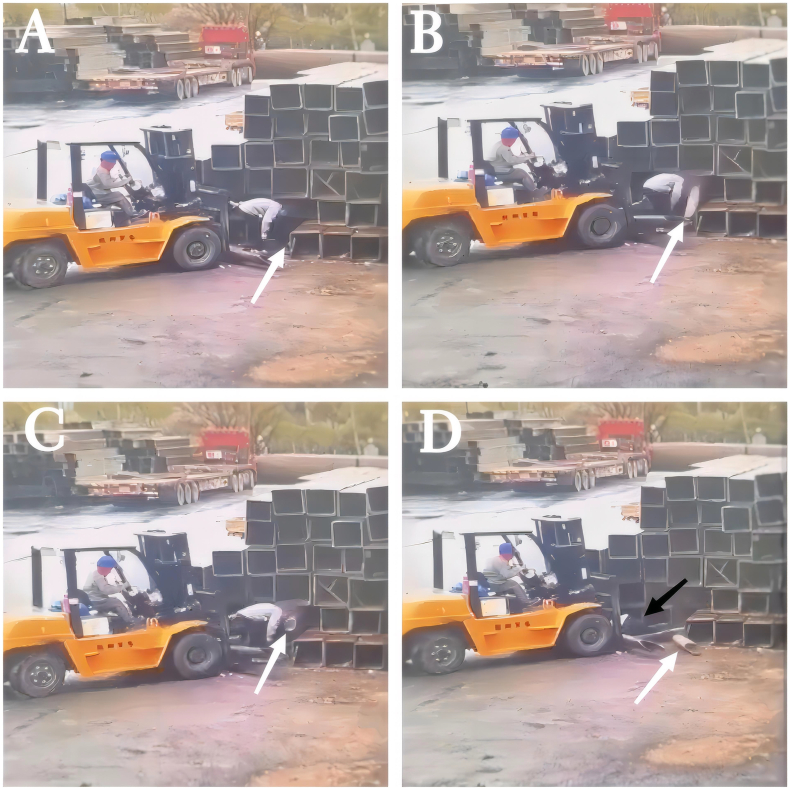
(A and B and C and D) Patient's injury scene, with a white arrow pointing to the steel pipe, and a black arrow pointing to the patient.

**Fig. 2:**
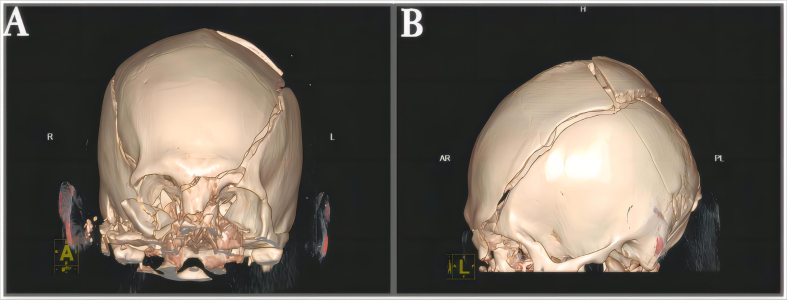
(A and B)The three-dimensional reconstructed image of the first head CT scan upon the patient's admission.

**Fig. 3:**
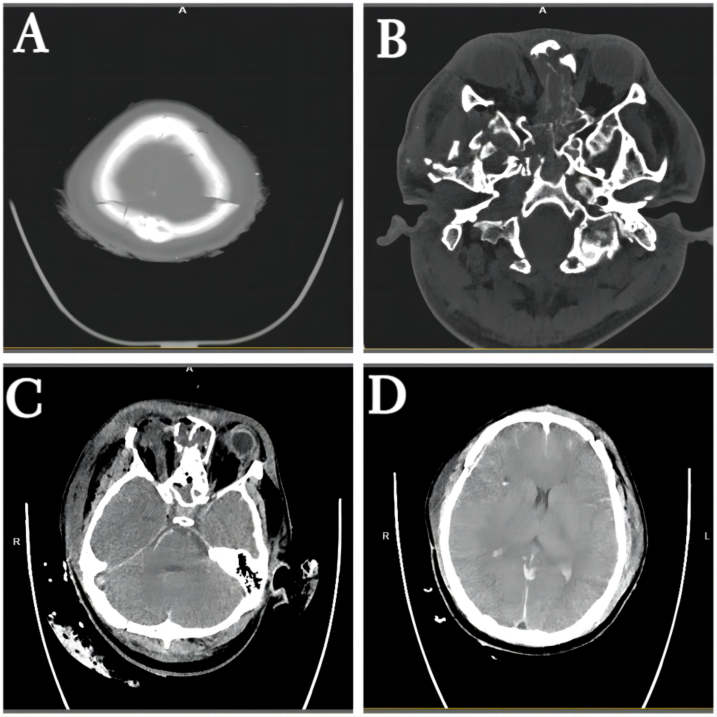
(A and B and C and D)The first head CT image upon the patient's admission.

**Fig. 4:**
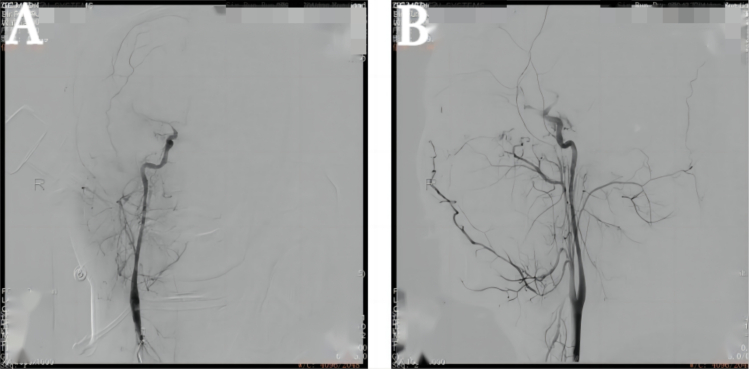
(A and B)The distal ends of multiple branches of the right external carotid artery show contrast agent accumulation.

**Fig. 5:**
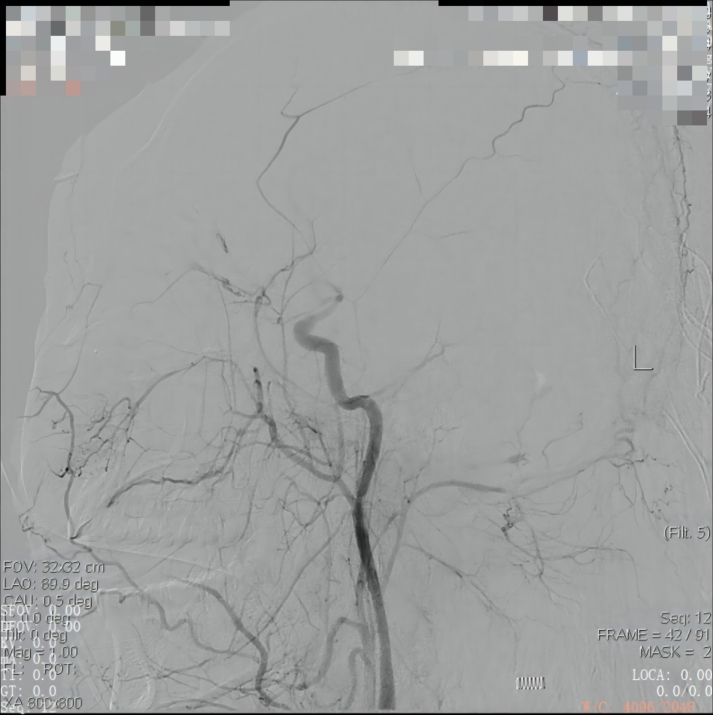
The right internal carotid artery shows a small carotid-cavernous fistula. There is contrast agent overflow observed in the distal end of the branches of the left external carotid artery (mainly superficial temporal artery and occipital artery).

**Fig. 6:**
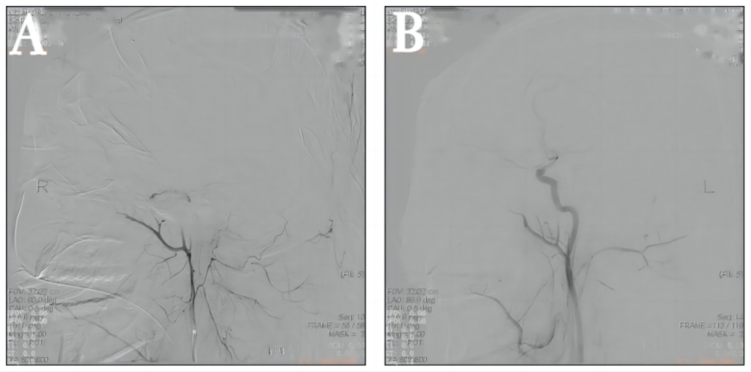
(A and B)Embolization performed by injecting gelatin sponge particles; post-procedure reimaging did not show clear signs of active bleeding.

**Fig. 7:**
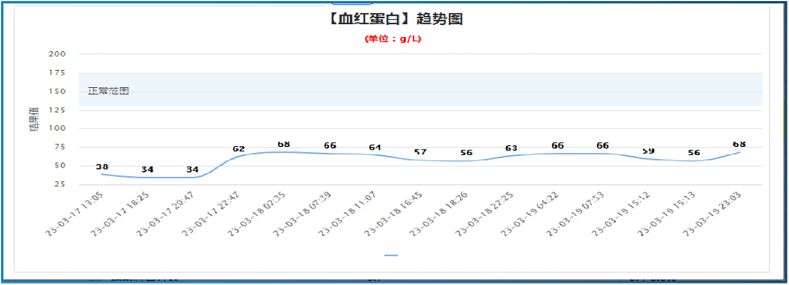
Patient's hemoglobin level trend chart.

**Fig. 8:**
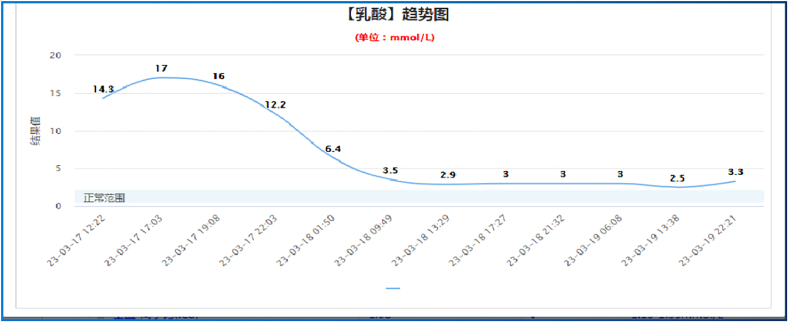
The patient's blood lactate level trend chart.

**Fig. 9:**
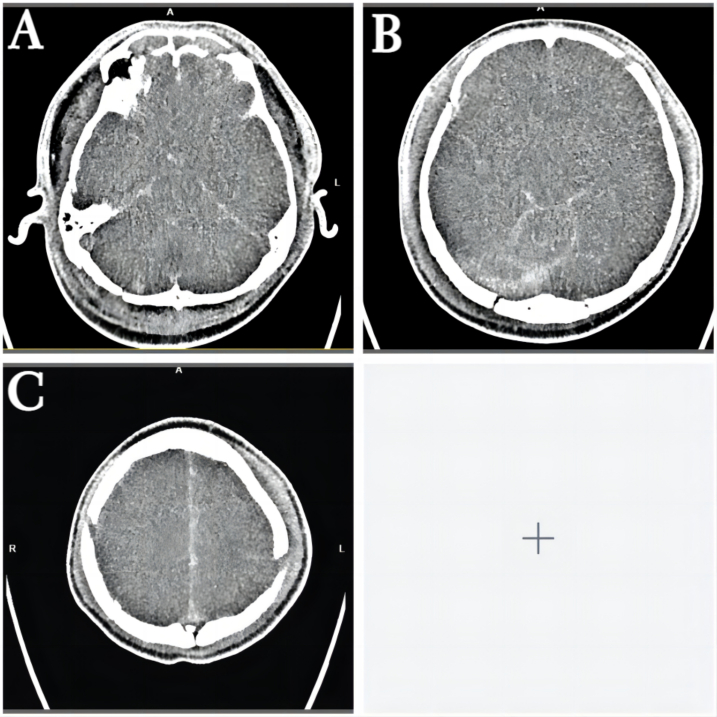
(A and B and C)Imaging of the patient's follow-up head CT on the 5th day of admission.

**Fig. 10:**
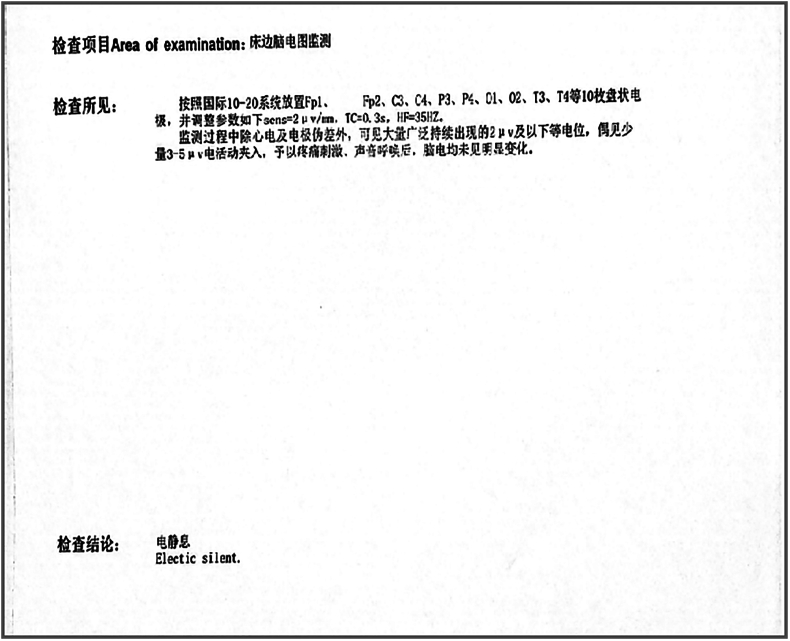
The patient's bedside electroencephalogram (EEG)and the report.

**Fig. 11:**
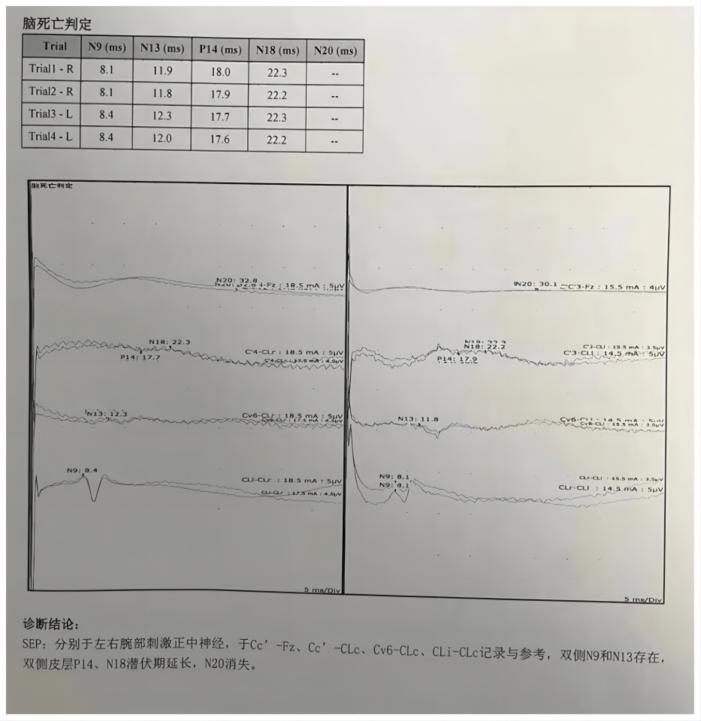
Short-latency somatosensory evoked potentials(SLSEP).

**Fig. 12:**
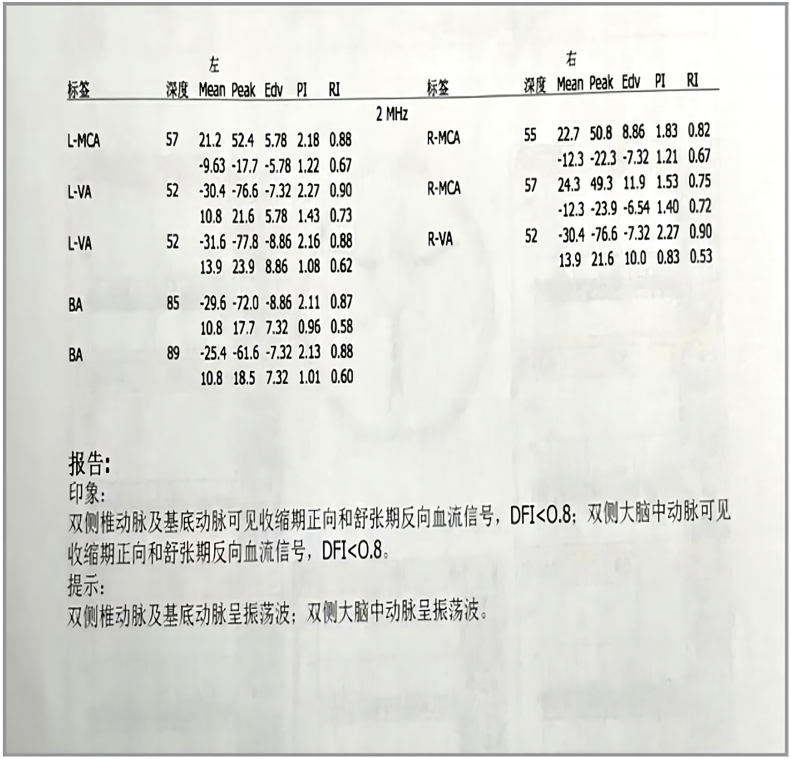
Transcranial Doppler (TCD) report.

A previously healthy 29-year-old patient, while at work, was struck in the head by a steel pipe launched by a forklift. The patient who lost consciousness immediately with multiple sites of active bleeding on the head and face was transported to the nearest secondary hospital by the emergency services (120) promptly.(Figs. 1). Upon arrival at the hospital's emergency department, the patient was comatose (GCS score of 3), with progressive bleeding from the head and face, a decreasing blood pressure, necessitating emergency tracheal intubation and mechanical ventilation. High doses of vasoactive drugs were administered to maintain blood pressure, along with tranexamic acid for hemostasis, blood transfusion, and fluid resuscitation. The CT result of the head revealed multiple skull fractures and brain contusions (Figs. 2 A,B and Figs. 3 A, B, C, D). Considering the critical condition, the patient received blood transfusion and was promptly transferred to a tertiary hospital one hour after injury. Upon arrival at the tertiary hospital's emergency department, physical examination indicated: heart rate of 25 beats per minute, blood pressure of 50/40 mmHg, unmeasurable SPO2, multiple skin lacerations on the head and face, durative staxis, periorbital ecchymosis, bleeding from the nose and mouth, GCS score of 3, tracheal intubation, cervical immobilization, fixed and dilated bilateral pupils measuring 5 mm with absent light reflexes. The patient's heart rate progressively decreased, prompting immediate cardiopulmonary resuscitation. Simultaneously, intravenous administration of epinephrine for cardiac support, sodium bicarbonate for correcting acidosis, emergency blood transfusion (at a ratio of red blood cells, plasma, and platelets in a 1:1:1 proportion), supplementation of fibrinogen, cryoprecipitate, crystalloid fluids, maintaining warmth, and wound closure of the head was provided as part of the treatment. A negative FAST bedside ultrasound was performed, arterial blood gas analysis indicated pH 7.16, PO2 69.3 mmHg, PCO2 19.8 mmHg, base excess (BEB) -19.8 mmol/l, lactate (LAC) 14.3 mmol/l, hemoglobin (Hb) 38 g/l, prothrombin time (PT) >120 s, activated partial thromboplastin time (APTT) >180 s, D-dimer >20 mg/l, fibrinogen (FG) <0.4 g/l. Based on the mechanism of injury, symptoms, and ancillary investigations, a tentative diagnosis included severe head trauma, traumatic cerebral hemorrhage, traumatic brain herniation, subarachnoid hemorrhage, multiple skull fractures, traumatic coagulopathy, hemorrhagic shock, and blunt cerebrovascular injury. Considering blunt cerebrovascular injury with active bleeding, immediate contact was made with the interventional radiology department for a planned DSA. After 27 min of resuscitation, the patient regained spontaneous heart rate and respiration, palpable arterial pulsations, and electrocardiogram showed sinus rhythm. The DSA procedure was performed successfully, revealing active bleeding mainly from multiple branches of the right external carotid artery (including the ascending pharyngeal artery, maxillary artery, superficial temporal artery, facial artery) (Figs. 4 A, B). A small amount of carotid-cavernous fistula was observed in the right internal carotid artery, and active bleeding was indicated in the branches of the left external carotid artery (mainly superficial temporal artery and occipital artery) (Figs. 5). Blood supply within the intracranial branches of both internal carotid arteries was sluggish. Microcatheters were selectively placed in the above-mentioned main arterial vessels and branches, and gelatin sponge particles were used for embolization. Post-procedure reimaging did not show clear signs of active bleeding(Figs. 6 A, B). The patient was then admitted to the Intensive Care Unit for monitoring and treatment. Hemoglobin levels stabilized after embolization (Figs. 7), and blood lactate levels progressively decreased (Figs. 8). On the 5th day of admission, a follow-up head CT revealed significant brain tissue swelling, intraventricular and subarachnoid hemorrhage (Figs. 9 A, B, C). On the 6th day of hospitalization, the patient was declared brain dead by specialist clinicians (Figs. 10 and Figs. 11 and Figs. 12).

## 3 Clinical discussion

Blunt cerebrovascular injury encompasses both ischemic and hemorrhagic conditions [5].Due to its low incidence and atypical symptoms, BCVI is often missed, yet its consequences can be catastrophic, particularly in Grade V injuries which are frequently fatal and require immediate hemorrhage control. Patients with certain clinical symptoms or signs following blunt trauma should promptly undergo imaging studies to assess vascular injury: potential pulsatile bleeding from the neck, mouth, nose, or ear; expanding neck hematomas; bruits in patients under 50 years old; focal or unilateral neurological deficits inconsistent with head CT or MRI findings; CT or MRI indicating cerebral infarction [6]. Although enhanced CT or digital subtraction arteriography (DSA) are sensitive tools for identifying cerebrovascular injury, they are not routinely used clinically. Therefore, careful and meticulous evaluation of the patient's condition is necessary to avoid misdiagnosis and ensure timely intervention. Delay in diagnosis results in higher mortality rates and more severe ischemia and hypoxia of brain cells, leading to worse prognoses.

## 4 Conclusion

Cases of active bleeding in cerebrovascular injuries following trauma are rare but associated with low survival rates. Using DSA in combination with endovascular treatment in emergencies helps reduce the mortality rate of these highly fatal injuries. It is imperative to train medical professionals at all levels in understanding these conditions and the relevant techniques of endovascular treatment, which could significantly impact the treatment outcomes for these patients. Medical facilities unable to perform this technique should promptly transfer such cases to higher-level medical institutions after evaluating their eligibility for transfer and assessment.

## 5 Methods section

The work has been reported in line with the SCARE criteria [7].

## Sources of funding

No fund support.

## Ethical approval

This research study has been given ethical approval.

## Consent

Written informed consent was obtained from the patient's family for publication of this case report and accompanying images. A copy of the written consent is available for review by the Editor-in-Chief of this journal on request.

## Author contribution

Dr.Xizhu Huang,the attending physicians from department of Emergency, contributions to the paper, study design, data analysis, writing the paper. And thanks for the contribution from Dr.Xianglei Kong and Dr. Wenlong Fan, the attending physicians from department of radiology to provide the radiology examination data. And thanks for the contribution from Dr. Yongxia CAI, the attending physicians from department of Emergency, for searching data collection.

## Guarantor

Dr.Xizhu Huang.

## Registration of research studies

N/A

## Conflict of interest statement

The authors declare that there is no conflict of interest for the publication of this article.

## References

[2] Biffl, W.L., et al., Treatment-related outcomes from blunt cerebrovascular injuries: importance of routine follow-up arteriography. Ann. Surg., 2002. 235(5): p. 699–706; discussion 706–7. 10.1097/01.SLA.0000027174.01008.A0. PMC1422496 11981216

[3] Berne J.D. (2001). The high morbidity of blunt cerebrovascular injury in an unscreened population: more evidence of the need for mandatory screening protocols. J. Am. Coll. Surg..

[4] Miller, P.R., et al., Prospective screening for blunt cerebrovascular injuries: analysis of diagnostic modalities and outcomes. Ann. Surg., 2002. 236(3): p. 386–93; discussion 393–5. 10.1097/01.SLA.0000027174.01008.A0. PMC1422592 12192325

[5] Biffl W.L. (1999). Blunt carotid arterial injuries: implications of a new grading scale. J. Trauma.

[6] Burlew C.C. (2012). Blunt cerebrovascular injuries: redefining screening criteria in the era of noninvasive diagnosis. J. Trauma Acute Care Surg..

[7] Sohrabi C., Mathew G., Maria N., Kerwan A., Franchi T., Agha R.A. (2023). The SCARE 2023 guideline: updating consensus surgical case report (SCARE) guidelines. Int J Surg Lond Engl..

